# Methyl Salicylate Enhances Flavonoid Biosynthesis in Tea Leaves by Stimulating the Phenylpropanoid Pathway

**DOI:** 10.3390/molecules24020362

**Published:** 2019-01-21

**Authors:** Xin Li, Li-Ping Zhang, Lan Zhang, Peng Yan, Golam Jalal Ahammed, Wen-Yan Han

**Affiliations:** 1 Key Laboratory of Tea Quality and Safety Control, Ministry of Agriculture, Tea Research Institute, Chinese Academy of Agricultural Sciences, 9 Meiling Road, Hangzhou 310008, China; lixin@tricaas.com (X.L.); lpzhang8263@163.com (L.-P.Z.); zhanglan@tricaas.com (L.Z.); yanpengzn@tricaas.com (P.Y.); 2 College of Forestry, Henan University of Science and Technology, Luoyang 471023, China

**Keywords:** salicylic acid, flavonoids, phenylpropanoid pathway, phenylalanine ammonia-lyase (PAL), tea quality

## Abstract

The phytohormone salicylic acid (SA) is a secondary metabolite that regulates plant growth, development and responses to stress. However, the role of SA in the biosynthesis of flavonoids (a large class of secondary metabolites) in tea (*Camellia sinensis* L.) remains largely unknown. Here, we show that exogenous methyl salicylate (MeSA, the methyl ester of SA) increased flavonoid concentration in tea leaves in a dose-dependent manner. While a moderate concentration of MeSA (1 mM) resulted in the highest increase in flavonoid concentration, a high concentration of MeSA (5 mM) decreased flavonoid concentration in tea leaves. A time-course of flavonoid concentration following 1 mM MeSA application showed that flavonoid concentration peaked at 2 days after treatment and then gradually declined, reaching a concentration lower than that of control after 6 days. Consistent with the time course of flavonoid concentration, MeSA enhanced the activity of phenylalanine ammonia-lyase (PAL, a key enzyme for the biosynthesis of flavonoids) as early as 12 h after the treatment, which peaked after 1 day and then gradually declined upto 6 days. qRT-PCR analysis of the genes involved in flavonoid biosynthesis revealed that exogenous MeSA upregulated the expression of genes such as *CsPAL*, *CsC4H*, *Cs4CL*, *CsCHS*, *CsCHI*, *CsF3H*, *CsDFR*, *CsANS* and *CsUFGT* in tea leaves. These results suggest a role for MeSA in modulating the flavonoid biosynthesis in green tea leaves, which might have potential implications in manipulating the tea quality and stress tolerance in tea plants.

## 1. Introduction

Tea is the most popular non-alcoholic beverage consumed across the seven continents [1]. However, tea cultivation is mostly confined to Asia and Africa [2]. Green tea, which is produced from the young shoots of *Camellia sinensis* (L.) Kuntze has a range of human health benefits, such as anti-cancer, anti-inflammatory, anti-allergic and anti-obesity effects [3,4]. Flavonoids are the major antioxidative constituents in tea leaves that function against cancer, cardiovascular disease, diabetes, obesity and metabolic syndrome [5,6]. Flavan-3-ol type flavonoids, i.e., catechin compounds, impart the characteristic astringency and bitterness to green tea infusions [7,8]. Catechins act as precursors of theaflavins and thearubigins that are developed during black tea processing [2]. Therefore, flavonoids are considered as an important group of constituents that largely determine the quality of tea. 

In plants, flavonoids biosynthesis occurs in the endoplasmic reticulum, from where they are transported to different cellular compartments for specific functions [9,10]. Flavonoids are a large and diverse group of secondary metabolites that are synthesized through a specific branch of phenylpropanoid pathway [11,12]. Phenylalanine is the initial substrate of this pathway, which is deaminated by the catalysis of phenylalanine ammonia-lyase (PAL), the first and a rate-limiting enzyme that regulates overall carbon flux into phenylpropanoid metabolism due to its unique metabolic position [13]. Stress conditions trigger flavonoid biosynthesis, possibly to scavenge overproduced reactive oxygen species (ROS) [14]. In addition to the ability of flavonoids to scavenge ROS, role of flavonoids as “signaling molecules” or “developmental regulators” have been revealed in plants [15]. Some flavonoids interact with hormone signaling and regulate plant organ development [16].

Plant hormones are important endogenous signal molecules that regulate a plethora of metabolic processes and responses to stress [17]. Studies on the effect of plant hormones on flavonoid concentration in tea revealed a largely hormone-specific response [11]. For example, exogenous gibberellins (GA_3_) and abscisic acid (ABA) decrease flavonoid concentration [13,18], whereas brassnosteroid application enhances endogenous flavonoid levels in tea leaves [7,8]. Salicylic acid (SA) is an important plant hormone that primarily functions in the immune response [19]. Nonetheless, roles of SA in plant growth, development and stress tolerance have also been revealed. Like flavonoids, SA is also synthesized from phenylalanine via cinnamic acid [20]. Since both SA and flavonoids are phenylpropanoid derivatives and have antioxidant capacity, SA application affects flavonoid biosynthesis [20]. Nevertheless, the effect of methyl salicylate (MeSA, the methyl ester of SA) on flavonoid biosynthesis in tea is largely unknown. In the present study, we show that exogenous MeSA could increase flavonoid concentration in tea leaves, which is associated with the MeSA-induced enhanced activity of PAL and transcriptional upregulation of genes involved in flavonoid biosynthesis. Our results suggest that MeSA has a significant stimulatory effect on flavonoid biosynthetic pathway which could be exploited to manipulate tea quality and stress tolerance. 

## 2. Results

### 2.1. MeSA Increases Flavonoid Content in a Concentration-Dependent Manner

**Figure 1:**
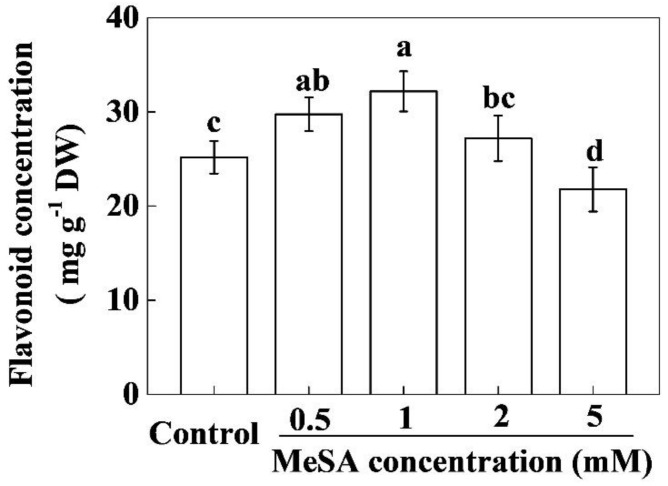
Effect of different concentrations of methyl salicylate (MeSA) on flavonoid concentrations in tea leaves. Tea bushes were sprayed with different concentrations of MeSA (0, 0.5, 1, 2 and 5 mM) and samples were harvested after 1 day for the biochemical analysis. The data of flavonoid concentrations were expressed as the mean values ± SD, *n* = 6. Mean denoted by the different letters indicate significant differences between the treatments (*p* < 0.05).

To assess whether exogenous MeSA could alter flavonoid levels in tea leaves, we analyzed the flavonoid concentration after application of different concentrations of MeSA. The results showed that moderate concentrations of MeSA (0.5–1 mM) increased flavonoid concentrations in tea leaves, reaching the highest value with 1 mM MeSA (27.78% compared to control). However, higher concentrations of MeSA either had no effect or negatively influenced flavonoid concentration in tea leaves (Figure 1). A time course of flavonoid concentrations after 1 mM MeSA treatment showed that flavonoid concentration began to rise after MeSA application, reaching the maximum level at 2 days post-treatment. For instance, flavonoid concentration increased by 30.64% and 29.91% after 1 and 2 days post treatment with MeSA, respectively as compared with that of control. Afterward, flavonoid concentrations gradually decreased.

**Figure 2:**
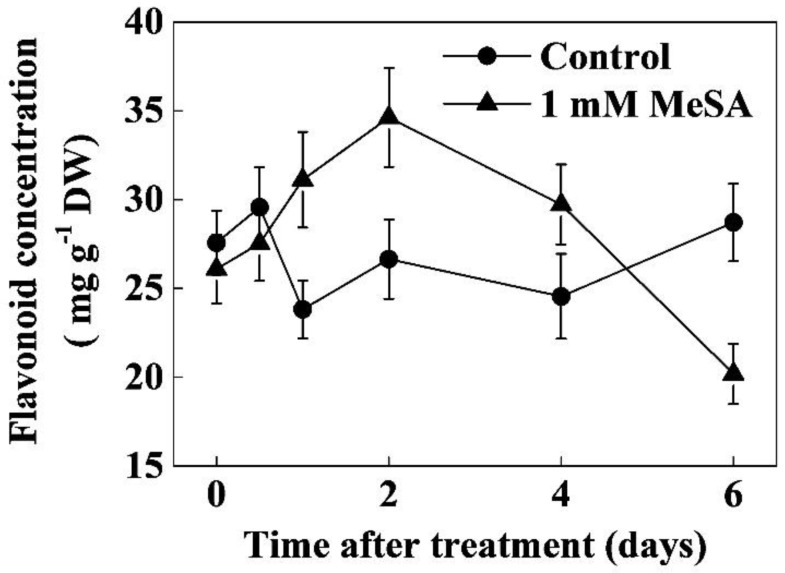
Time course of flavonoid concentration as influenced by exogenous methyl salicylate (MeSA) as foliar spray. Leaf samples were harvested at indicated time-points following foliar spray with 1 mM MeSA. The data of flavonoid concentrations were expressed as the mean values ± SD, *n* = 6.

After 6 days, the level of flavonoids in MeSA-treated leaves decreased by 29.69% compared with that of control (Figure 2). We also analyzed the total and individual catechin concentrations at 2 days post treatment with 1 mM MeSA. The results showed that MeSA treatment significantly increased the total catechin concentration in tea leaves which was attributed to significant increases in (−)-epigallocatechin-3-gallate (EGCG), epicatechins gallate (ECG) and (−)-epigallocatechin (EGC). However, (−)-catechin (C) and epicatechins (EC) concentrations were not altered by MeSA treatment (Supplementary Figure S1).

### 2.2. Changes in PAL Activity after MeSA Application

**Figure 3:**
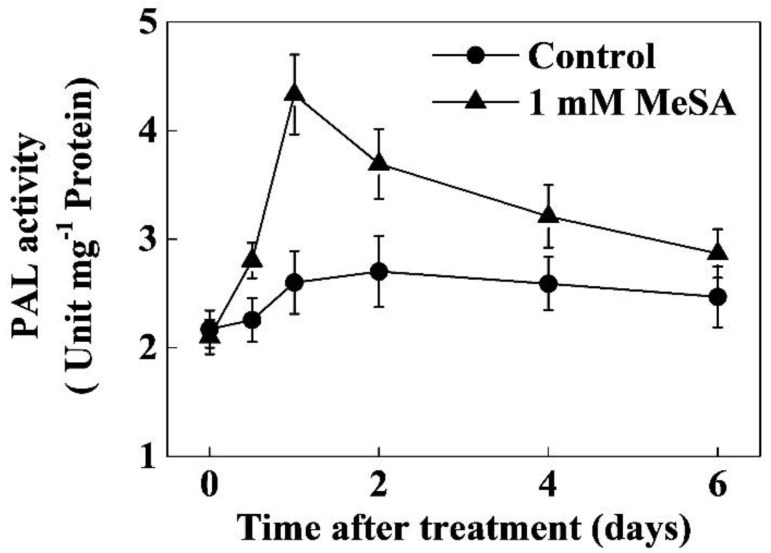
Time course of the phenylalanine ammonia-lyase (PAL) activity as influenced by exogenous methyl salicylate (MeSA). Tea bushes were sprayed with 1 mM MeSA. The data of PAL activity were expressed as the mean values ± SD, *n* = 6.

Next, we analyzed the activity of PAL, the first enzyme of the phenylpropanoid pathway. Consistent with the time-course of flavonoid concentration, the PAL activity increased gradually after MeSA treatment, which peaked at 1 day after MeSA treatment (Figure 3). More precisely, the PAL activity increased by 66.58% compared with that of control at 1 day after MeSA treatment. Then the PAL activity in MeSA-treated leaves declined, but remained 36.70% and 23.90% higher than that of control at 2 and 4 days post treatment, respectively, before reaching the level close to the control at 6 days post treatment (Figure 3). Although the PAL activity was differentially regulated by exogenous MeSA at different time points, total soluble protein concentration was not remarkably affected by MeSA in tea leaves (Supplementary Figure S2). 

### 2.3. MeSA Modulates Transcript Levels of Flavonoid Biosynthetic Genes

**Figure 4:**
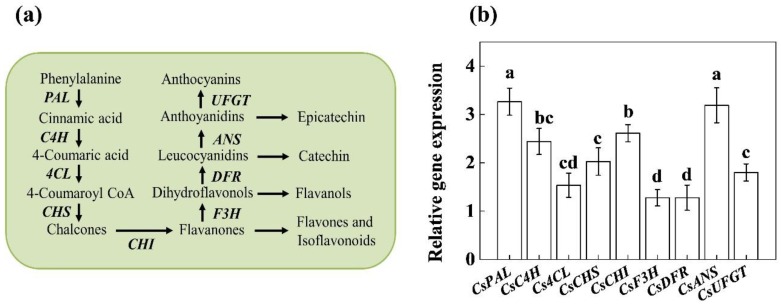
Effect of methyl salicylate on flavonoid biosynthetic pathway in tea leaves. (**a**) Nine key genes involved in flavonoid biosynthesis are marked in bold letters in italic. Adopted and redrawn from Li et al. [12]. (**b**) the expression of flavonoid biosynthetic genes in tea leaves. Transcript levels of the genes were analyzed by qRT-PCR using gene-specific primer pairs (Supplementary Table S1) and expressed as fold change relative to the control. Tea bushes were sprayed with 1 mM methyl salicylate (MeSA) and samples were harvested after 1 day for qRT-PCR assay. The data are mean of 3 biological replicates. Bars denoted by the different letters indicate significant differences between different expression levels of flavonoid biosynthetic genes (*p* < 0.05). *PHENYLALANINE AMMONIA-LYASE (PAL)*, *CINNAMATE 4-HYDROXYLASE (C4H)*, *p-COUMARATE:COA LIGASE (4CL)*, *CHALCONE SYNTHASE (CHS)*, *CHALCONE ISOMERASE (CHI), FLAVANONE 3-HYDROXYLASE (F3H)*, *DIHYDROFLAVONOL 4-REDUCTASE (DFR)*, *ANTHOCYANIDIN SYNTHASE (ANS)* and *UDP- GLUCOSE FLAVONOID 3-O-GLUCOSYL TRANSFERASE (UFGT).*

To clarify whether the MeSA-induced changes in flavonoid concentration were attributed to concomitant changes in flavonoids biosynthesis, we analyzed the transcript levels of nine key genes involved in flavonoid biosynthetic pathway (Figure 4a), such as *PHENYLALANINE AMMONIA-LYASE (PAL)*, *CINNAMATE 4-HYDROXYLASE (C4H)*, *p-COUMARATE:COA LIGASE (4CL)*, *CHALCONE SYNTHASE (CHS)*, *CHALCONE ISOMERASE (CHI)*, *FLAVANONE 3-HYDROXYLASE (F3H*), *DIHYDROFLAVONOL 4-REDUCTASE (DFR)*, *ANTHOCYANIDIN SYNTHASE (ANS)* and *UDP- GLUCOSE FLAVONOID 3-O-GLUCOSYL TRANSFERASE (UFGT)*. 

qRT-PCR analysis showed that exogenous MeSA caused approximately 3-fold increase in transcript levels of *CsPAL* which was more or less consistent with the activity of PAL (Figure 3 or Figure 4b). Similarly, MeSA treatment increased the transcript levels of the rest of the genes, such as *CsC4H* (2.4-fold), *Cs4CL* (1.5-fold), *CsCHS* (2.1-fold), *CsCHI* (2.6-fold), *CsF3H* (1.3-fold), *CsDFR* (1.2-fold), *CsANS* (3.2-fold) and *CsUFGT* (1.8-fold) compared to that of control. These findings suggest that exogenous MeSA differentially regulates transcription of different genes in flavonoid biosynthetic pathway to increase flavonoid concentrations in tea leaves.

## 3. Discussion

Flavonoids are the key secondary metabolites that contribute to the value of plant products from agronomic, industrial, and nutritional points of view [11]. Particularly, in case of green tea, flavonoids impact not only the tea taste (astringency) but also the health benefits and economic value [21]. Different hormone signaling pathways differentially regulate flavonoid biosynthesis, which is highly species-specific [8,11,13,18,22]. The role of methyl salycilate (MeSA) in flavonoid biosynthesis in tea leaves remained largely unknown. Here we found that an optimal dose of MeSA could increase flavonoid concentration in tea leaves (Figure 1). Time course analysis revealed that MeSA-induced enhancement in flavonoid concentration peaked after 1–2 days, which was consistent with the increased activity of PAL in tea leaves (Figure 2 and Figure 3). Gene expression analysis relating to flavonoid biosynthesis suggested that exogenously applied MeSA stimulated transcriptional machinery causing differential upregulation in the respective transcripts (Figure 4). Therefore, MeSA application at 1 day prior to harvesting may potentially increase flavonoid concentrations in green tea. 

Flavonoids are low molecular weight antioxidants that serve as major defense compounds against abiotic and biotic stressors in plants [11,15,20,23]. It is believed that flavonoids play a major role in scavenging ROS when antioxidant enzymes are depleted under stress [9,23]. In the presence of light, SA treatment significantly increases flavonoid content in *Ginkgo biloba* leaves [24], which is in agreement with the current study (Figure 1). Similarly, MeJA increases flavonoid accumulation in citrus leaves in the first 12 h after treatment [25], which is slightly different from our observation, possibly due to the differences in elicitors and plant species. Furthermore, we found that exogenous MeSA rapidly and transiently increased PAL activity in tea leaves, which was consistent with the concentrations of flavonoids (Figure 2 and Figure 3). PAL is the key enzyme in the first step of the phenylpropanoid pathway which regulates biosynthesis of thousands of phenylpropanoids [18]. PAL links the secondary metabolism to primary metabolism and maintains the metabolic flow of carbon into the phenylpropanoid pathway in plants [13]. In the present study, exogenous MeSA increased both the activity and transcription of PAL gene (*CsPAL*) in tea leaves (Figure 3 and Figure 4). MeSA-induced promotion in PAL activity perhaps stimulated subsequent reactions in phenylpropanoid pathway, leading to an enhanced production of phenylpropanoid derivatives including flavonoids (Figure 1, Figure 2 and Figure 3). Our results are consistent with a previous report that showed that root application of SA in hydroponics increases PAL activity and specific flavonoid content in wheat leaves [20]. 

In the flavonoid biosynthesis pathway, *CHS*, *CHI* and *F3H* are termed as early biosynthetic genes, whereas downstream genes such as *DFR*, *ANS* and *UFGT* are named late biosynthetic genes [11,26]. In the current study, exogenous MeSA upregulated the transcript levels of both early and late biosynthetic genes in tea leaves (Figure 4). Different hormones differentially modulate the expression of flavonoid biosynthesis genes [8,11,22,26]. For instance, ABA and GA_3_ down-regulate *CsPAL, CsC4H, CsF3H* and *CsANR* expression, leading to a decreased catechin content in tea leaves [13,18]. However, exogenous brassinosteroids increase *CsPAL* expression with concomitant increase in flavonoid concentration in tea leaves [7,8]. In *Vitis vinifera*, PAL expression reaches the peak at 3 h post-elicitation with MeJA, which gradually declines returning to the basal levels at 48 h post-treatment in the presence of light [26]. Consistent with this, jasmonic acid and brassinosteroids also enhance transcript levels of late biosynthetic genes and myeloblastosis (MYB) transcription factor in Arabidopsis seedlings [22]. Notably, at transcriptional levels, flavonoid biosynthesis is regulated by the MYB transcription factors [11], in which SA can induce MYBs to regulate specific phenylpropanoid (capsaicinoid) biosynthesis [27]. Therefore, such regulatory mechanism in response to exogenous MeSA might also function in tea leaves, however, this interpretation demands further in-depth investigation in current direction. 

To sum up, in the current study, we found that: (1) a moderate dose of exogenous MeSA (1 mM) increased flavonoid concentration, but a high dose of MeSA (5 mM) showed an opposite effect, (2) 1 mM MeSA appeared to be the best concentration to simulate endogenous flavonoid accumulation in tea leaves, (3) MeSA-induced increase in flavonoid concentration was maximized after 1–2 day followed by gradual decline over time, (4) MeSA-induced increase in flavonoid concentration was associated with the simultaneous increase in the activity of PAL and transcription of early and late biosynthetic genes in flavonoid biosynthetic pathway. These results suggest that 1–2 day prior application of MeSA can be an effective method to manipulate flavonoid concentration in tea leaves. Moreover, MeSA-induced flavonoid biosynthesis may enhance plant tolerance to biotic and/or abiotic stressors.

## 4. Materials and Methods

### 4.1. Plant Materials and Growth Conditions

In the current experiment, “Longjing 43” tea (*Camellia sinensis* L.) cultivar was used as plant materials and the study was conducted at tea garden of the Tea Research Institute, Chinese Academy of Agricultural Sciences, Hangzhou, China. Foliar portion of tea bushes was sprayed with a series of freshly prepared methyl salicylate (MeSA) solutions (0.5, 1, 2 and 5 mM). Each treatment comprises 4 replicates, while each replicate represents an area of 10 m^2^ consisting of 20 tea bushes.

### 4.2. Determination of Flavonoid Concentration

For the determination of flavonoid concentration, leaf samples were extracted in 70% ethanol (*v*/*v*) at 100 °C, and the concentration of total flavonoids was measured in the aqueous extract following AlCl_3_ method as described previously [28]. Absorbance at 510 nm was recorded for the determination and rutin was used as the standard. Total and individual catechin concentrations in tea leaves were determined with a Waters 590 HPLC system (Waters Corp., Milford, MA, USA) equipped with a Thermo Scientific^TM^ Hypersil^TM^ ODS-2 C18 column (5 μm particle size, 4.6 mm × 250 mm, Thermo Fisher Scientific Inc., Waltham, MA, USA) at 280 nm as previously described [12].

### 4.3. Assay of Phenylalanine Ammonia-Lyase (PAL) Enzyme Activity

A tea sample (0.3 g) was homogenized in 3 mL 50 mM potassium phosphate buffer (pH 8.8, containing 2 mM EDTA, 2% PVPP, and 0.1% mercaptoethanol). The resulting homogenate was centrifuged at 15,000 rpm for 20 min at 4 °C and the crude enzyme extract was obtained as the supernatant. l-phenylalanine was used as substrate to assay the PAL activity based on the yield of cinnamic acid. The change in absorbance at 290 nm was monitored to determine the PAL activity as described previously [29].

### 4.4. Total RNA Extraction and Gene Expression Analysis

For gene expression analysis, tea leaf samples were collected at 1 day after MeSA treatment and immediately frozen into liquid nitrogen and kept at −80 °C until RNA isolation. Total RNA was extracted using an RNA extraction kit (Tiangen Biotech, Beijing, China) and reverse transcribed using a ReverTra Ace qPCR RT kit (Toyobo, Osaka, Japan) following the manufacturer’s instructions. Gene-specific primers were designed based on their cDNA sequences (Supplementary Table S1). Quantitative real-time PCR (qRT-PCR) was performed on the ABI 7500 Real-Time PCR system (Applied Biosystems, Foster City, CA, USA) using SYBR Green PCR Master Mix (Takara, Shiga, Japan). The qRT-PCR cycling conditions were as follows: 95 °C for 30 s, and 40 cycles of 95 °C for 5 s and 60 °C for 34 s. Relative gene expression was calculated according to previously described method using *CsPTB* as the internal reference gene [30]. 

### 4.5. Statistical Analysis

The data were statistically analyzed using SAS 8.1 software package (SAS Institute Inc., Cary, NC, USA). Differences between treatments means were separated by the Tukey’ test at a significance level of *p* < 0.05. 

## Supplementary Materials

The following is available online. Table S1: Primers used for qRT-PCR assays in tea leaves, Figure S1: Total and individual catechins concentrations in tea leaves as influenced by exogenous methyl salicylate as foliar spray, Figure S2: Time course of total soluble protein concentrations as influenced by exogenous methyl salicylate.
