# Supplementary material for: Methyl Salicylate Enhances Flavonoid Biosynthesis in Tea Leaves by Stimulating the Phenylpropanoid Pathway
Source: Molecules. 2019 Jan 21;24(2):362. doi: 10.3390/molecules24020362 (PMC6359712; doi:10.3390/molecules24020362)
Supplement: Supplementary file 1 [file molecules-24-00362-s001.pdf]

Supplemental Table S1. Primers used for real time qRT-PCR assays.

Genes	Functional annotation		Primer pairs
CsPTB	*POLYPYRIMIDINE TRACT-BINDING PROTEIN*	GAAC0105249 8.1	F: 5'- TGACCAAGCACACTCCACACTATCG-3'<br>R: 5'- TGCCCCCTTATCATCATCCACAA-3'
CsPAL	*PHENYLALANINE AMMONIA-LYASE*	D26596	F: 5'-GAATGCCGGTCTTATCCACT-3'<br>R: 5'-CGGTGAACACCTTGTCAAAC-3'
CsC4H	*CINNAMATE 4-HYDROXYLASE*	AY641731	F: 5'-CGAGAGGTTCTTGGAAGAGG-3'<br>R: 5'-AGAATTGGCAGAGCAAGGAT-3'
Cs4CL	*P-COUMARATE:COA LIGASE*	DQ194356	F: 5'-GGAGGTTATCCTGGACCTCA-3'<br>R: 5'-GGCAAGCCTTGTAGTGTGAA-3'
CsCHS	*CHALCONE SYNTHASE*	AY169403	F: 5'-GGCAATCAAAGAATGGGG-3'<br>R: 5'-ATGGGCGAAGACCGAGT-3'
CsCHI	*CHALCONE ISOMERASE*	DQ904329	F: 5'-CACAAAGAAGATTATGGGTGAAG-3'<br>R: 5'-CAAACCTAGAAGTTGCCAAGAGT-3'
CsF3H	*FLAVANONE 3-HYDROXYLASE*	AY641730	F: 5'-CTACTCAAGATGGCCCGACAA-3'<br>R: 5'-ACAACACCTCCAGCAACTTGC-3'

CsDFR	*DIHYDROFLAVONOL 4-REDUCTASE*	AB018685	F: 5’-ATTGGCAGAGAAAGCAGCAT-3’
			R: 5’-GTGATTAGGCTTGGTGGGAA-3’
CsANS	*ANTHOCYANIDIN SYNTHASE*	AY830416	F: 5’-GGCCACAAGTGCCTACAATTG-3’
			R: 5’-CCCATGATTCACCAAATGCA-3’
CsUFGT	*UDP- GLUCOSE FLAVONOID 3-O-GLUCOSYL TRANSFERASE*	GH618818	F: 5’-GGCAAGAAGCTAATAGGGTCGTT-3’
			R: 5’-TTGTATCATTCGGAAGTGgTGGG-3’

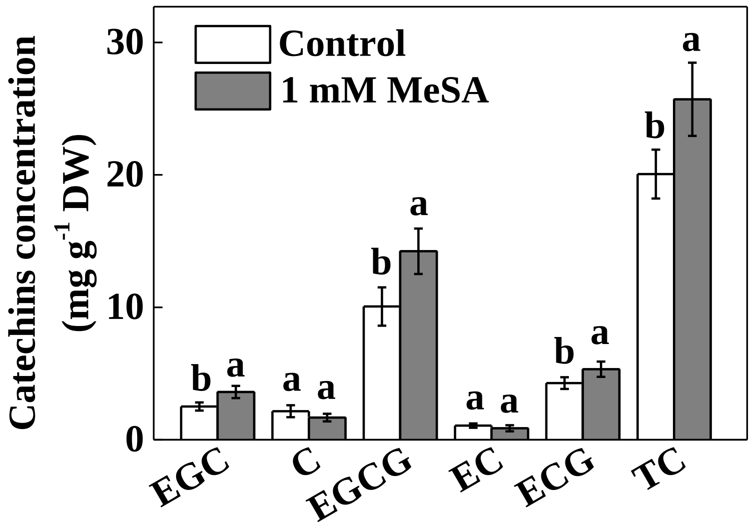

Supplementary Figure S1. Total and individual catechins concentrations in tea leaves as influenced by exogenous methyl salicylate (MeSA) as foliar spray. Leaf samples for catechins determination were harvested at 2 day post-treatment with 1 mM MeSA. The data of catechins concentrations were expressed as the mean values ±SD, n=6. Means denoted by the different letters indicate significant differences between the treatments ($P<0.05$). (-)-epigallocatechin, EGC; (-)-catechin, C; (-)-epigallocatechin-3-gallate, EGCG; epicatechins, EC; epicatechins gallate, ECG; total catechins, TC.

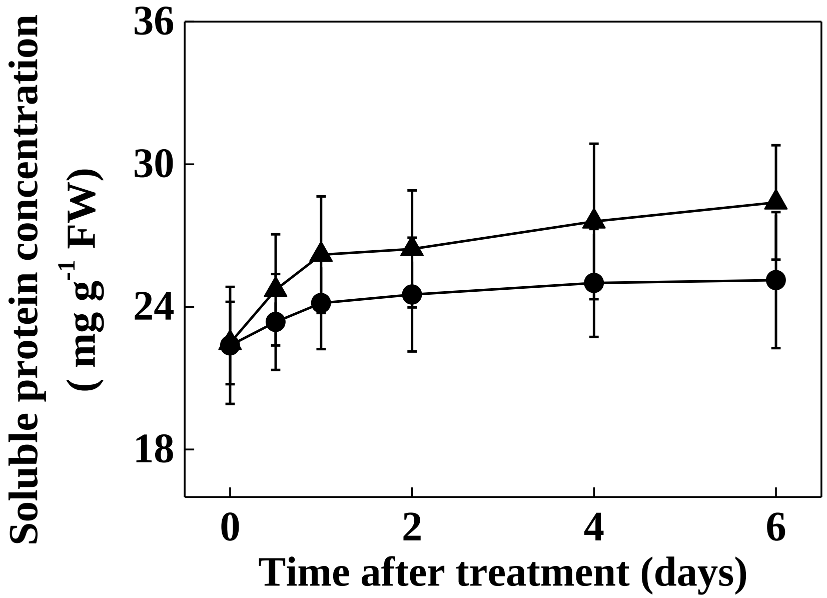

Supplementary Figure S2. Time course of total soluble protein concentrations as influenced by exogenous methyl salicylate (MeSA). Tea bushes were sprayed with 1 mM MeSA. Line graphs with solid circle (●) and triangle (▲) represent data of control and MeSA treatment, respectively. The data were expressed as the mean values ±SD, n=6.
